# The interferon-induced antiviral protein PML (TRIM19) promotes the restriction and transcriptional silencing of lentiviruses in a context-specific, isoform-specific fashion

**DOI:** 10.1186/s12977-016-0253-1

**Published:** 2016-03-22

**Authors:** Nasser Masroori, Natacha Merindol, Lionel Berthoux

**Affiliations:** Laboratory of Retrovirology, Department of Medical Biology and BioMed Research Group, Université du Québec à Trois-Rivières, Trois-Rivières, QC Canada

**Keywords:** HIV-1, Lentivirus, PML, Restriction factor, Interferon, Latency, Innate immunity

## Abstract

**Background:**

The promyelocytic leukemia (PML) protein, a type I interferon (IFN-I)-induced gene product and a member of the tripartite motif (TRIM) family, modulates the transcriptional activity of viruses belonging to various families. Whether PML has an impact on the replication of HIV-1 has not been fully addressed, but recent studies point to its possible involvement in the restriction of HIV-1 in human cells and in the maintenance of transcriptional latency in human cell lines in which HIV-1 is constitutively repressed. We investigated further the restriction of HIV-1 and a related lentivirus, SIV_mac_, by PML in murine cells and in a lymphocytic human cell line. In particular, we studied the relevance of PML to IFN-I-mediated inhibition and the role of individual human isoforms.

**Results:**

We demonstrate that both human PML (hPML) and murine PML (mPML) inhibit the early post-entry stages of the replication of HIV-1 and a related lentivirus, SIV_mac_. In addition, HIV-1 was transcriptionally silenced by mPML and by hPML isoforms I, II, IV and VI in MEFs. This PML-mediated transcriptional repression was attenuated in presence of the histone deacetylase inhibitor SAHA. In contrast, depletion of PML had no effect on HIV-1 gene expression in a human T cell line. PML was found to contribute to the inhibition of HIV-1 by IFN-I. Specifically, IFN-α and IFN-β treatments of MEFs enhanced the PML-dependent inhibition of HIV-1 early replication stages.

**Conclusions:**

We show that PML can inhibit HIV-1 and other lentiviruses as part of the IFN-I-mediated response. The restriction takes place at two distinct steps, i.e. reverse transcription and transcription, and in an isoform-specific, cellular context-specific fashion. Our results support a model in which PML activates innate immune antilentiviral effectors. These data are relevant to the development of latency reversal-inducing pharmacological agents, since PML was previously proposed as a pharmacological target for such inhibitors. This study also has implications for the development of murine models of HIV-1.

**Electronic supplementary material:**

The online version of this article (doi:10.1186/s12977-016-0253-1) contains supplementary material, which is available to authorized users.

## Background

In mammals, many effectors are involved in the innate immune response to pathogens, including viruses. Of particular interest are restriction factors that are members of the tripartite motif (TRIM) protein superfamily. Several of the TRIM superfamily members are upregulated by IFN-I, suggesting that they might be involved in antiviral innate immunity (reviewed in [1]). PML, also known as TRIM19, is a member of this family of proteins. PML was initially identified as part of a hybrid protein that also contains retinoic acid receptor α (RARα) and that causes acute promyelocytic leukemia [2–4]. PML is expressed in all cell lines tested [5] and localizes to the nucleus; it is found both in the nucleoplasm and in association with a nuclear multiprotein structure called the nuclear body (NB) [6, 7]. In addition to PML, NBs include several other proteins, but the integrity of this structure depends on the presence of PML [8]. The transcription of some PML NB proteins, including PML and Sp100, is upregulated by interferons [5, 9, 10] and contributes to cellular defense mechanisms [11].

The interactions between PML (or PML NBs) and viruses have been well documented. Soon after the discovery of PML NBs, Maul and colleagues showed that herpes simplex virus type 1 (HSV-1) causes the cellular redistribution of PML from PML NBs [12]. Further investigations demonstrated that the HSV-1 immediate-early (IE) gene product ICP0 localizes to and disrupts PML NBs, resulting in an increase in viral gene expression [13]. In human cytomegalovirus (HCMV)-infected cells, the PML NB-associated protein Daxx (Death domain-associated protein) silences viral immediate-early gene expression, but this antiviral mechanism is counteracted by the HCMV protein pp71 [14, 15]. It has also been reported that constitutive overexpression of PML in mouse cells induces resistance to infection by RNA viruses, such as vesicular stomatitis virus (VSV) and influenza A [16]. Furthermore, IFN-induced overexpression of PML in wild-type (WT) mouse embryonic fibroblasts (MEFs) represses the transcription of human foamy virus (HFV), a retrovirus, by forming a complex with the HFV transactivator, Tas, thereby preventing the direct binding of Tas to viral DNA [17]. Accordingly, this inhibitory mechanism is not observed in PML knockout (KO) cells. In contrast to the antiviral activities often associated with PML, it was recently shown that depleting PML reduces the production of infectious hepatitis C virus particles, indicating that PML may enhance virus particle production [18]. Likewise, establishment of human papillomavirus (HPV) is enhanced by PML expression in the early part of the life cycle [19]. Whether PML modulates the permissiveness to HIV-1 and other lentiviruses has been controversial [20, 21], but recent reports have converged toward an inhibitory role for PML [22, 23].

Although antiretroviral therapy (ART) is capable of decreasing HIV-1 viral load to levels below the limit of detection in many patients, the virus is not eliminated and interruption of ART almost always leads to a rapid viral rebound and progression to AIDS [24]. HIV-1 is capable of establishing a state of latent infection when activated CD4^+^ T-cells (the major target of HIV-1) become infected and then revert back to a resting memory state [25–27]. These infected resting T-cells show low or absent viral gene expression and provide a viral reservoir that is protected from immune clearance and ART (reviewed in [28]). Current strategies to eradicate this reservoir aim at reactivating the latent proviruses by using various agents such as the histone deacetylase (HDAC) inhibitor suberoylanilide hydroxamic acid (SAHA; Vorinostat) [29] and the acetaldehyde dehydrogenase inhibitor disulfiram [30], often combined with protein kinase C agonists [31]. Despite the current interest in pharmacological strategies to disrupt the quiescence of latent proviruses, the mechanism by which HIV-1 persists in the presence of ART is not well understood. In a recent study, the proximity of HIV-1 proviruses to PML NBs was found to correlate with the extent of HIV-1 gene expression silencing in a T cell-based HIV-1 latency model. Accordingly, PML degradation resulted in the activation of viral transcription following proviral displacement from PML NBs [32]. Here, we examined the involvement of PML in the restriction of HIV-1 in human and murine cells. Our results provide evidence that PML is a component of the innate immune response to lentiviruses and may participate in HIV-1 gene silencing and latency.

## Results

### PML depletion increases the susceptibility of human T cells to lentivirus infection

**Fig. 1:**
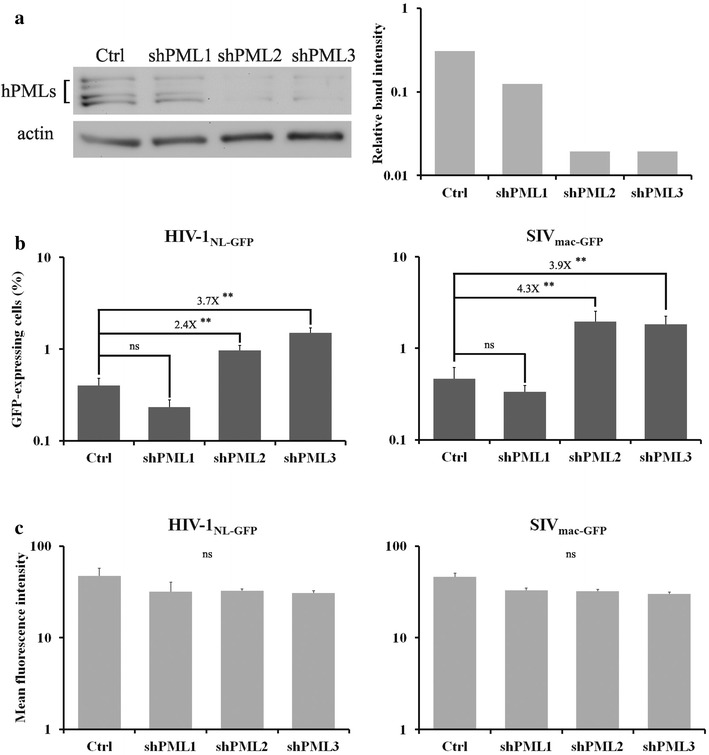
PML-mediated restriction of HIV-1 and SIV_mac_ infection in SupT1 cells. **a** WB analysis of human SupT1 cells stably transduced with shPMLs. Cells were stably transduced with either the control shRNA targeting luciferase or with shRNAs targeting all hPML isoforms. PML expression levels were analyzed by WB using a polyclonal antibody (*upper panel*). The same blot was reprobed with an anti-actin antibody as a loading control. The *graph* on the *right* shows the ratios of PML compared to actin following densitometry analysis. **b** Effects of shRNA-mediated depletion of hPML on HIV-1 and SIV_mac_ infectivity. The cells stably expressing shPMLs or control shRNAs were infected with HIV-1_NL-GFP_ (*left*) or SIV_mac-GFP_ (*right*) (MOI of 0.1). Two days later, the percentages of infected cells were measured by FACS. The values represent the means of three independent experiments with standard deviations (**P < 0.01, two-tailed Student’s *t* test). **c** Effects of shRNA-mediated depletion of hPML on HIV-1 and SIV_mac_ LTR-driven GFP expression. GFP MFI values are shown for the experiments in **b** (*ns* non-significant in the two-tailed Student’s *t* test)

We first investigated the effect of endogenous hPML depletion on HIV-1 and SIV_mac_ infectivity in SupT1 cells, a human T lymphoblastoid cell line. The cells were stably transduced with lentiviral vectors expressing shRNAs targeting all hPML isoforms or expressing an shRNA against luciferase as a control, and conferring puromycin resistance. The untransduced cells were eliminated by puromycin treatment and PML knockdown was analyzed by WB (Fig. 1a). The results showed that both shPML2 and shPML3 efficiently decreased PML expression in SupT1 cells, whereas shPML1 had no significant effect. We next infected the 4 SupT1 pools with low viral doses of VSV protein G (VSV-G)-pseudotyped, green fluorescent protein (GFP)-expressing lentiviral vectors based on HIV-1 strain NL43 (HIV-1_NL-GFP_) and simian immunodeficiency virus strain mac239 (SIV_mac-GFP_) for 2 days, followed by fluorescence-activated cell sorting (FACS) analysis. In these vectors, GFP is inserted in the Nef ORF and HIV-1 Env expression is disrupted [33, 34]. The results showed that PML depletion, mediated by shPML2 and shPML3, increased the percentage of cells infected with HIV-1_NL-GFP_ (2.4-fold and 3.7-fold, respectively), whereas shPML1 had no significant effect (Fig. 1b, left panel). Similarly, the permissiveness of SupT1 cells to SIV_mac-GFP_ was increased 4.3-fold and 3.9-fold by expression of PML shRNA2 and shRNA3, respectively, whereas shRNA1 had no effect (Fig. 1b, right panel).

GFP is routinely used as a reporter protein to study the activity of promoters; in particular, quantitation of GFP fluorescence intensity is a robust marker for expression levels, as it has been shown to directly correlate with mRNA levels in individual cells [35]. Recently, GFP fluorescence intensity was used to analyze the Cas9 nuclease-mediated knockout of latently integrated HIV-1 genomes in human cells [36]. GFP expression by the HIV-1-based vector HIV-1_NL-GFP_, which is used in our study, is under the control of a natural 5′-LTR that acts as an enhancer and a promoter. This allowed us to investigate whether hPML interferes with HIV-1 gene expression by quantifying GFP mean fluorescence intensity (MFI) in infected cells using FACS. None of the shRNAs used had any significant effect on the GFP MFI following infection with HIV-1_NL-GFP_ or SIV_mac-GFP_ (Fig. 1c). Thus, PML restricts the early stages of HIV-1 and SIV_mac_ infection but does not affect viral gene expression in SupT1 cells.

### PML confers resistance to infection of murine cells by lentiviruses

**Fig. 2:**
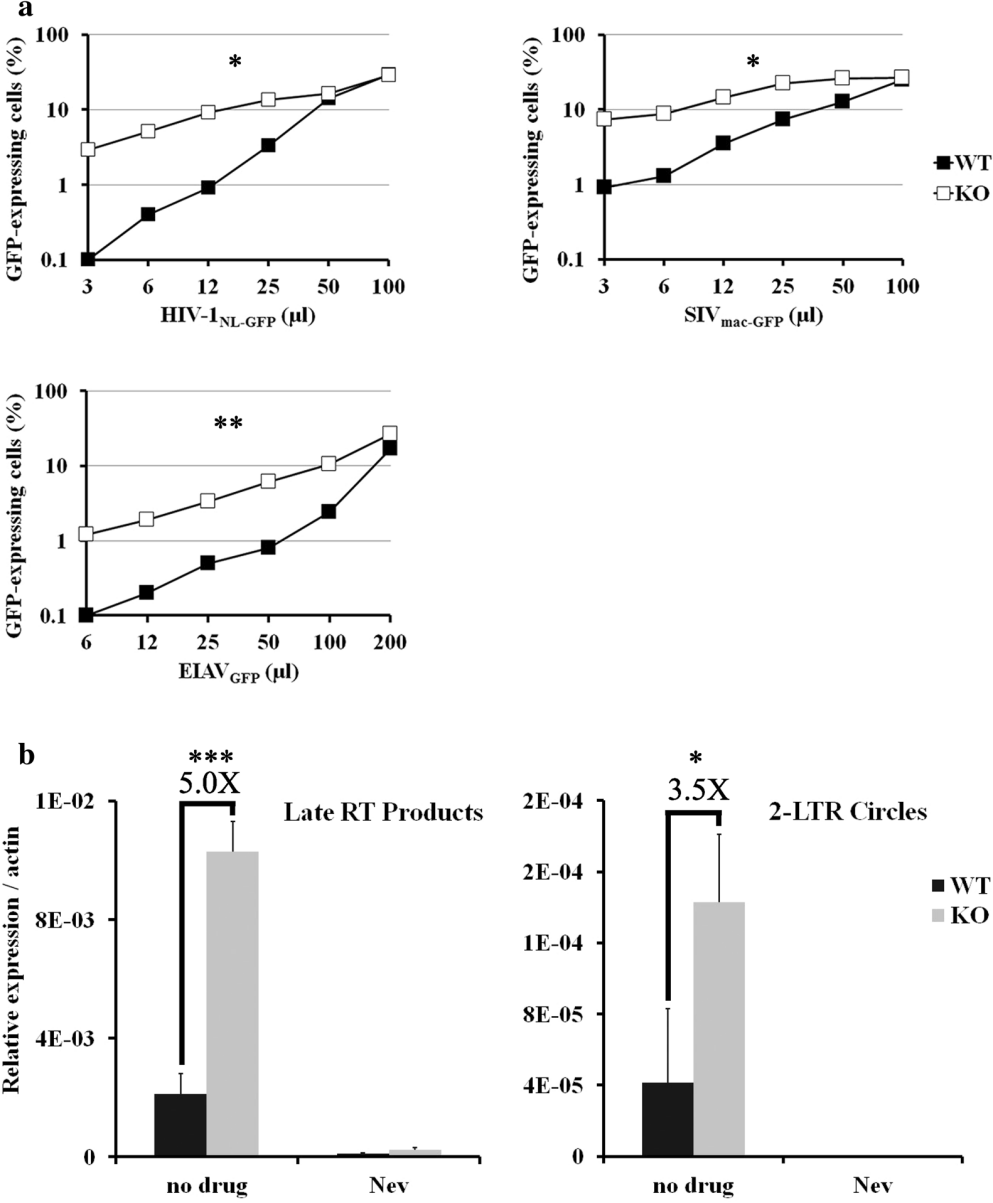
Murine PML confers resistance to infection with lentiviruses. **a** Dose-dependent analysis of retrovirus infectivity. WT and PML-KO MEF cells were infected with increasing doses of HIV-1_NL-GFP_, SIV_mac-GFP_ and EIAV_GFP_. The percentage of infected (GFP-expressing) cells was measured 2 days later by FACS. Shown is one experiment representative of ≥3 independent experiments that yielded comparable results (*P < 0.05; **P < 0.01, one-tailed paired Student’s *t* test). **b** Effects of PML on the early stages of HIV-1 replication. WT and PML-KO MEF cells were infected with HIV-1_NL-GFP_ at a low MOI (0.01 as measured on CRFK cells, see “Methods” section). Total cellular DNA was extracted 6 and 24 h post-infection and subjected to qPCR of HIV-1 late reverse transcription products and 2-LTR circles. Data are shown as relative viral products levels compared to actin. An RT inhibitor (nevirapine, Nev) was included as a control to show the absence of contaminating DNA. The values represent the means of three independent experiments with standard deviations (*P < 0.05; ***P < 0.001, two-tailed Student’s *t* test)

Restriction factors such as TRIM5α, apolipoprotein B mRNA editing enzyme, catalytic polypeptide-like 3G (APOBEC3G) and Tetherin often function in a species-specific, virus-specific fashion [37–39]. In order to analyze the antiretroviral potential of PML in a non-human context, PML-KO MEFs [40] and corresponding WT cells were challenged with increasing doses of HIV-1_NL-GFP_, SIV_mac-GFP_ and a GFP-expressing vector based on equine infectious anemia virus (EIAV_GFP_). The percentage of infected (GFP-positive) cells was then measured by FACS. We found that MEF cells were up to 30 times more permissive to infection by the HIV-1 vector in the absence of PML (Fig. 2a). Similarly, the infectivity of the SIV_mac_ and EIAV vectors was increased in PML-KO cells by up to 8-fold and 12-fold, respectively. This PML-dependent restriction phenotype decreased at higher virus doses (Fig. 2a), suggesting the presence of a saturation effect previously seen with TRIM5α [41], whereby large amounts of incoming retroviral cores “soak up” the restriction factor, resulting in attenuated or abrogated restriction. These data suggest that mPML is involved in a restriction mechanism targeting the early stages of infection by non-cognate lentiviruses. We used quantitative PCR (qPCR) to investigate the effects of PML on HIV-1 DNA synthesis and nuclear import, two early infection steps frequently affected by previously discovered restriction factors. When WT and PML-KO MEFs were infected with identical amounts of HIV-1_NL-GFP_, we observed ~5-fold more reverse transcribed DNA in the PML-KO cells (Fig. 2b). We also observed significantly more 2-LTR circles (a marker of nuclear import) in PML-KO cells (Fig. 2b). However, the effect of PML on 2-LTR circle levels was not greater than its effect on total reverse transcribed DNA, suggesting that the PML-dependent restriction of HIV-1 in MEFs affects mainly the reverse transcription step, consistent with recent findings from other groups [22, 23].

### PML promotes the down-regulation of HIV-1 LTR-driven GFP expression in MEFs

**Fig. 3:**
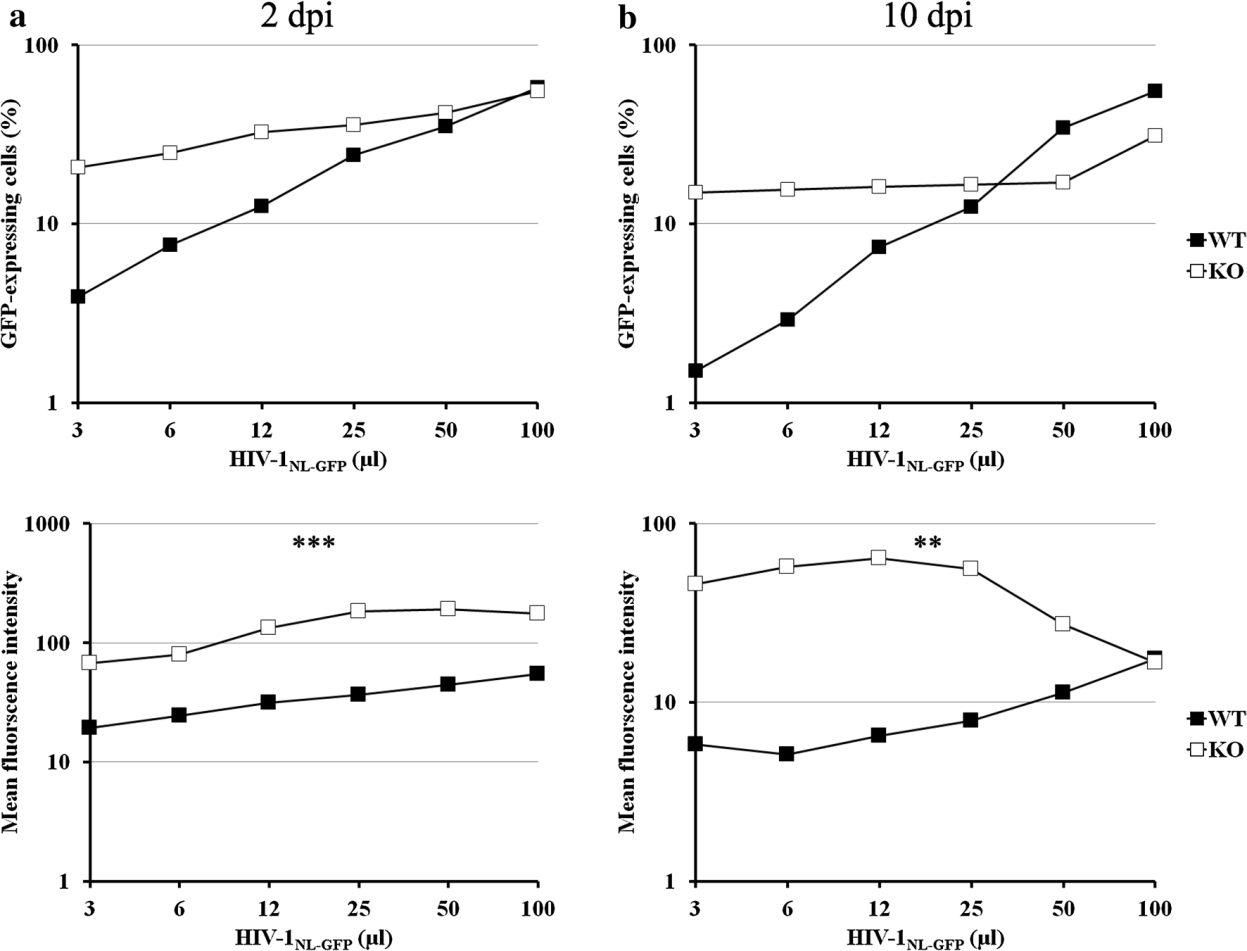
mPML knockout increases HIV-1 LTR-driven GFP expression. PML-KO and WT MEFs were infected with increasing doses of HIV-1_NL-GFP_ and cells were maintained in culture for 2 days (**a**) or 10 days (**b**), followed by FACS analysis. The percentage of infected (GFP-expressing) cells and the mean fluorescence intensity (MFI) were measured at each time point (*top* and *bottom panels*, respectively) (**P < 0.01; ***P < 0.001; one-tailed paired Student’s *t* test)

MEF WT and PML-KO cells were infected with increasing doses of HIV-1_NL-GFP_, as described above, followed by FACS. The percentage of infected cells and MFI (within the GFP+ population) were measured at 2 and 10 days post infection (dpi) (Fig. 3a, b, respectively). Performing the analyses at 10 dpi ensured that any GFP detected would have been expressed from integrated proviral DNA [42]. We found that PML knockout resulted in not only an increase in the percentage of GFP-expressing cells, but also an increase in the GFP MFI in these infected cells. Similar to what we observed in Fig. 2a, the effects of mPML knockout on viral infectivity were greatest when a low dose of virus was used and were abrogated at high virus doses (>25 µl of HIV-1_NL-GFP_ in this experiment). In contrast, the effects of mPML knockout on the GFP MFI were relatively more constant across multiple doses of virus (~4-fold increase at 2 dpi, ~10-fold at 10 dpi). At 10 dpi, however, we observed a decrease in GFP MFI at the two highest virus doses used (50 and 100 µl) in PML-KO cells, perhaps reflecting the existence of an additional PML-independent mechanism of inhibition. Altogether, results from the experiments shown in Figs. 2 and 3 suggest that PML expression in MEFs is associated with at least two distinct HIV-1 restriction mechanisms; one takes place at early post-entry stages, whereas the second results in a decrease in LTR-driven gene expression. The first inhibitory mechanism can be abrogated at high virus doses, whereas the second is not inhibited at these high doses, supporting a model where these activities are independent from each other.

### Overexpression of mPML restores restriction of HIV-1 and SIV_mac_ in PML-KO MEFs

**Fig. 4:**
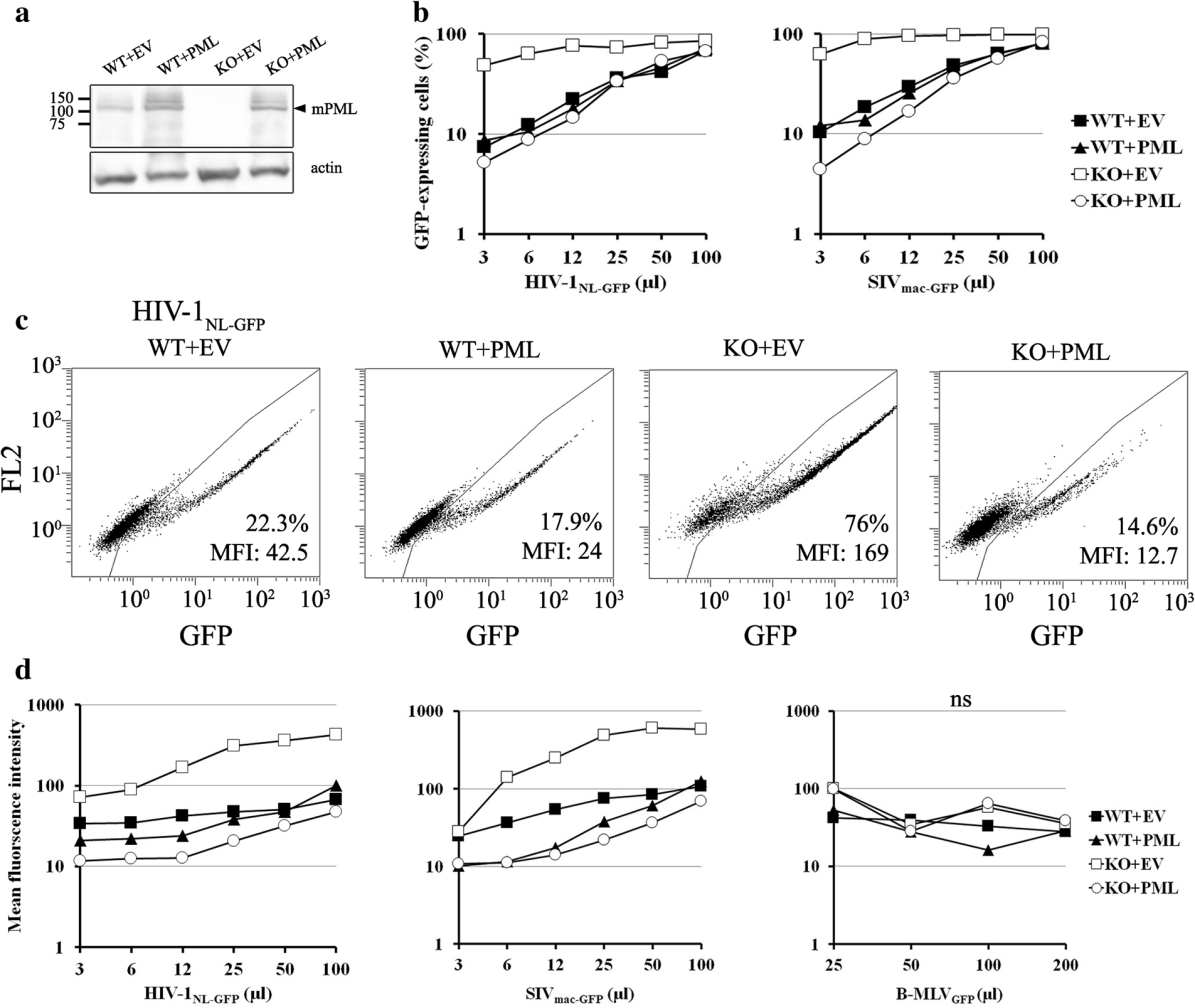
Expression of mPML in PML-KO MEFs restores restriction of HIV-1 and SIV_mac_. **a** Western blotting (WB) analysis of mPML overexpression in MEFs. PML cDNA was isolated from WT cells and transduced into both WT and PML-KO MEFs. The empty vector (EV) was transduced as a control. mPML expression was analyzed by WB of extracts from EV-transduced MEF-WT cells (WT + EV), mPML-transduced MEF-WT cells (WT + PML), EV-transduced PML-KO cells (KO + EV), and mPML-transduced PML-KO MEF cells (KO + PML). The WB was performed using an anti-mPML monoclonal antibody (*upper panel*) followed by an anti-actin antibody (*lower panel*) as a loading control. The *arrow points* to mPML, as judged from its expected size, whereas the heavier bands are presumably SUMOylated forms. The positions of the molecular size markers are indicated on the *left*. **b** Analysis of retrovirus infectivity in the transduced MEFs. The cells were infected with multiple doses of either HIV-1_NL-GFP_ or SIV_mac-GFP_, and the percentage of GFP-positive cells was measured at 2 dpi by FACS (P ≤ 0.001, one-tailed paired Student’s *t* test for KO + PML vs. KO). **c** FACS plots from transduced MEFs infected with HIV-1_NL-GFP_. WT and PML-KO MEF cells transduced with either EV or mPML were infected with HIV-1_NL-GFP_. The percentage of infected cells and mean fluorescence intensities determined at 2 dpi are indicated for each plot. **d** Down-regulation of LTR-driven GFP expression following overexpression of mPML in PML-KO MEFs. WT and PML-KO MEFs were stably transduced with either mPML or EV, as a control, then infected with multiple doses of HIV-1_NL-GFP_ (*left panel*), SIV_mac-GFP_ (*middle panel*) or B-MLV_GFP_ (*right panel*). The MFI was measured by FACS at 2 dpi (P < 0.01, one-tailed paired Student’s *t* test for KO + PML vs. KO after HIV-1_NL-GFP_ or SIV_mac-GFP_ infection)

Because the data reported above implicate mPML as a possible intrinsic defense factor against lentiviruses, we next examined whether its overexpression would restore the restriction of HIV-1 and SIV_mac_ in PML-KO MEFs. For this, we cloned the mPML cDNA from MEFs into the murine leukemia virus (MLV)-based vector pMIP [43] and transduced it in both WT and PML-KO MEFs, together with the empty vector (EV) as control. Our cloning strategy allowed for the isolation of both main isoforms (1 and 2) of mPML, but 3/3 sequenced clones corresponded to mPML isoform 2, which is the longest of the two (GenBank accession No. KJ650238). After puromycin selection of transduced cell populations, mPML expression was analyzed by Western blotting (WB). As shown in Fig. 4a, a band consistent with the expected size for mPML (110–120 kDa) was detected in the mPML-transduced cells, and a weaker band of the same size was seen in WT but not PML-KO MEFs. Additional bands corresponding to heavier proteins were also detected and could be SUMOylated forms. We then challenged the transduced cells with multiple doses of HIV-1_NL-GFP_ (Fig. 4b, left panel) and SIV_mac-GFP_ (Fig. 4b, right panel). Transduction of mPML into PML-KO MEFs decreased the infectivity of HIV-1_NL-GFP_ and SIV_mac-GFP_ by up to ~9-fold (Fig. 4b left panel) and ~14-fold (Fig. 4b right panel), respectively, similar to the levels seen in WT cells. In contrast to the PML-KO cells, overexpression of mPML in WT MEFs had no effect on infection with the HIV-1 and SIV_mac_ vectors. Like before, the magnitude of change in infectivity by PML knockout was greatest at the lowest viral doses. These results demonstrate that PML can inhibit the early stages of lentivirus infection in MEFs and suggest that endogenous mPML levels are sufficient to accomplish this function.

To provide further insights into the possible role of mPML in inhibiting lentiviral gene expression, we also measured the GFP MFI (Fig. 4c, d). As shown in representative FACS dot plots in Fig. 4c, overexpression of mPML in PML-KO MEF cells not only decreased HIV-1_NL-GFP_ infectivity from 76 to 14.6 % but also reduced the GFP MFI by 13.3-fold. In contrast, overexpression of mPML in WT MEF cells had only a small effect on the GFP MFI (less than 2-fold). Figure 4d summarizes the GFP MFI results obtained upon infection of mPML- or empty vector-transduced WT and PML-KO MEFs with HIV-1_NL-GFP_ and SIV_mac-GFP_. We found that overexpression of mPML in PML-KO MEFs strongly decreased the GFP MFI following infection by HIV-1_NL-GFP_ and SIV_mac-GFP_ (up to 13.3-fold and 22.3-fold, respectively). In contrast, overexpression of mPML in WT MEFs decreased GFP MFI by a much smaller magnitude following infection by HIV-1_NL-GFP_ and SIV_mac-GFP_ (up to 1.7-fold and 3.1-fold, respectively). As expected, the PML-induced reduction in GFP MFI was not dose dependent, thereby distinguishing the effects of mPML on infectivity and GFP MFI. As an additional control, we also infected the 4 cell pools with a GFP-expressing, “B-tropic” MLV-based vector [44], MLV_GFP_ (Fig. 4d, right panel) and similarly measured GFP expression levels. As anticipated, we found that the GFP MFI did not significantly vary in response to expression of mPML (either endogenous or exogenous). Collectively, the data shown in Figs. 2, 3 and 4 demonstrate that expression of mPML in murine cells inhibits both the infectivity and LTR-driven viral gene expression of non-murine lentiviruses.

### PML-dependent transcriptional silencing of HIV-1 in MEFs

**Fig. 5:**
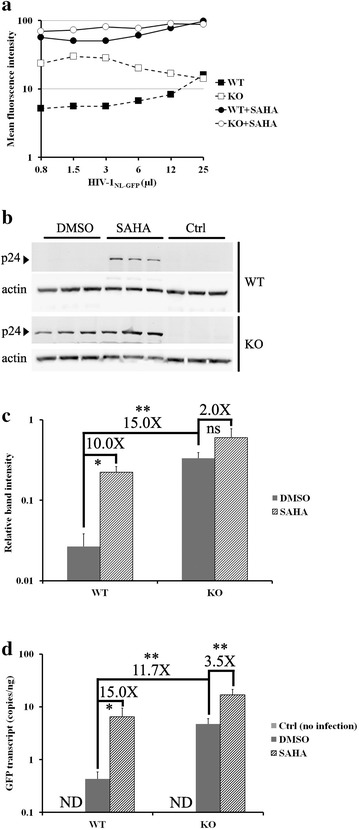
SAHA counteracts the PML-dependent inhibition of HIV-1 gene expression. **a** Effects of SAHA on HIV-1 LTR-driven GFP expression. PML-KO and WT MEFs were infected with increasing doses of HIV-1_NL-GFP_. Ten days later, the cells were treated with 5 µM SAHA or with DMSO for 48 h and the MFI was then measured by FACS (P = 0.0001, one-tailed paired Student’s *t* test for SAHA vs. DMSO treatment in WT cells). **b** Analysis of HIV-1 p24 expression levels. PML-KO and WT MEFs were infected in triplicate with HIV-1_NL-GFP_ at a CRFK MOI of 0.1 and then treated with either SAHA or DMSO at 10 dpi. Cellular lysates were prepared 48 h later and analyzed by WB using an anti-p24 antibody. Uninfected extracts were used as a negative control and actin was analyzed as a loading control. **c** The p24 and actin bands in the WB analysis shown in **b** were quantified by densitometry. The values represent the means of p24/actin ratios from the three data points for each condition with standard deviations (*P < 0.05; **P < 0.01, two-tailed Student’s *t* test). **d** qRT-PCR analysis of HIV-1 transcription. WT or PML-KO MEFs were infected with HIV-1_NL-GFP_ in triplicate. Ten days after infection, the cells were treated with either DMSO or SAHA for 48 h. Total RNA was purified from the cells and the level of GFP transcript was quantified by qRT-PCR. Total RNA from uninfected cells was used as a negative control. The values represent the means of three independent experiments with standard deviations (*P < 0.05; **P < 0.01, two-tailed Student’s *t* test). *ND* not detected

To further study the role of mPML in the regulation of the HIV-1 LTR-driven gene expression, we used SAHA, a HDAC inhibitor. HDACs act on histones within the nucleosome-bound promoter of HIV-1 to maintain proviral latency [45]. HDAC inhibition by SAHA leads to promoter expression and the escape of HIV-1 from transcriptional repression [29, 46]. Recently, SAHA was shown to affect the spatial distribution of hPML NBs [32]. We reasoned that if the HIV-1 LTR was repressed by mPML in MEFs in a fashion similar to its transcriptional repression in some human lymphocyte subpopulations [25], then SAHA would counteract the effects of mPML in MEFs. We infected WT and PML-KO MEFs with multiple doses of HIV-1_NL-GFP_ and maintained the cultures for 10 days. The cells were then treated with either 5 µM of SAHA or with DMSO as control for 48 h followed by FACS analysis of the GFP MFI, performed like before. As shown in Fig. 5a, the levels of GFP expression increased by up to ~11-fold in WT cells following treatment with SAHA, while a smaller increase (up to 3-fold) was observed in PML-KO cells. Therefore, SAHA counteracts the PML-mediated reduction in LTR-driven GFP expression in MEF cells, consistent with transcriptional repression of the LTRs as the underlying mechanism. Interestingly, at the highest dose of virus used, SAHA had the same effect on the GFP MFI in WT and PML-KO cells (Fig. 5a). This observation suggests that at high virus doses, HIV-1 LTR-driven gene expression may additionally become inhibited by a distinct mechanism independent of PML.

To insure that the inhibitory effect of PML on HIV-1 gene expression was not specific to the GFP reporter gene used in previous experiments, we also analyzed the expression of the HIV-1 p24 capsid protein in similar settings. WT and PML-KO MEFs were infected with HIV-1_NL-GFP_ in triplicate. 10 days later, the cells were treated or not with SAHA for 2 days and protein extracts were then analyzed by WB. As shown in Fig. 5b, c, p24 was barely detectable in DMSO-treated WT cells, whereas treatment with SAHA resulted in a 10-fold increase in p24 expression levels in these cells. In contrast, SAHA treatment caused only a 2-fold increase in p24 expression in PML-KO cells (Fig. 5c). Therefore, the results obtained in this experiment were consistent with those obtained for GFP.

To directly address whether the PML-dependent decrease in LTR-driven expression resulted from transcriptional repression, we used quantitative reverse transcription-PCR (qRT-PCR) to analyze the abundance of HIV-1 mRNA in MEF cells infected with HIV-1_NL-GFP_ exactly as in Fig. 5b. Levels of HIV-1 mRNA (analyzed using primers specific to the GFP coding sequence) were less than 1 copy per ng of total RNA in WT MEFs, but were 11.7 times higher in PML-KO cells (Fig. 5d). In response to the treatment of WT cells with SAHA, we observed a 15-fold increase in the levels of viral mRNA, compared to an increase of only 3.5-fold in the PML-KO cells. A second qPCR analysis was performed, this time normalized to actin transcription level (see Additional file 1). The results were consistent with those shown in Fig. 5d, as HIV-1 transcription was found to be 13.3-fold higher in the absence of mPML and SAHA specifically rescued HIV-1 transcription in WT MEFs. Taken together, these results provide strong evidence that mPML interferes with the HIV-1 transcription in MEFs.

### mPML-mediated restriction of lentiviruses does not require IFN-I, but mPML contributes to IFN-I-induced antiviral responses

**Fig. 6:**
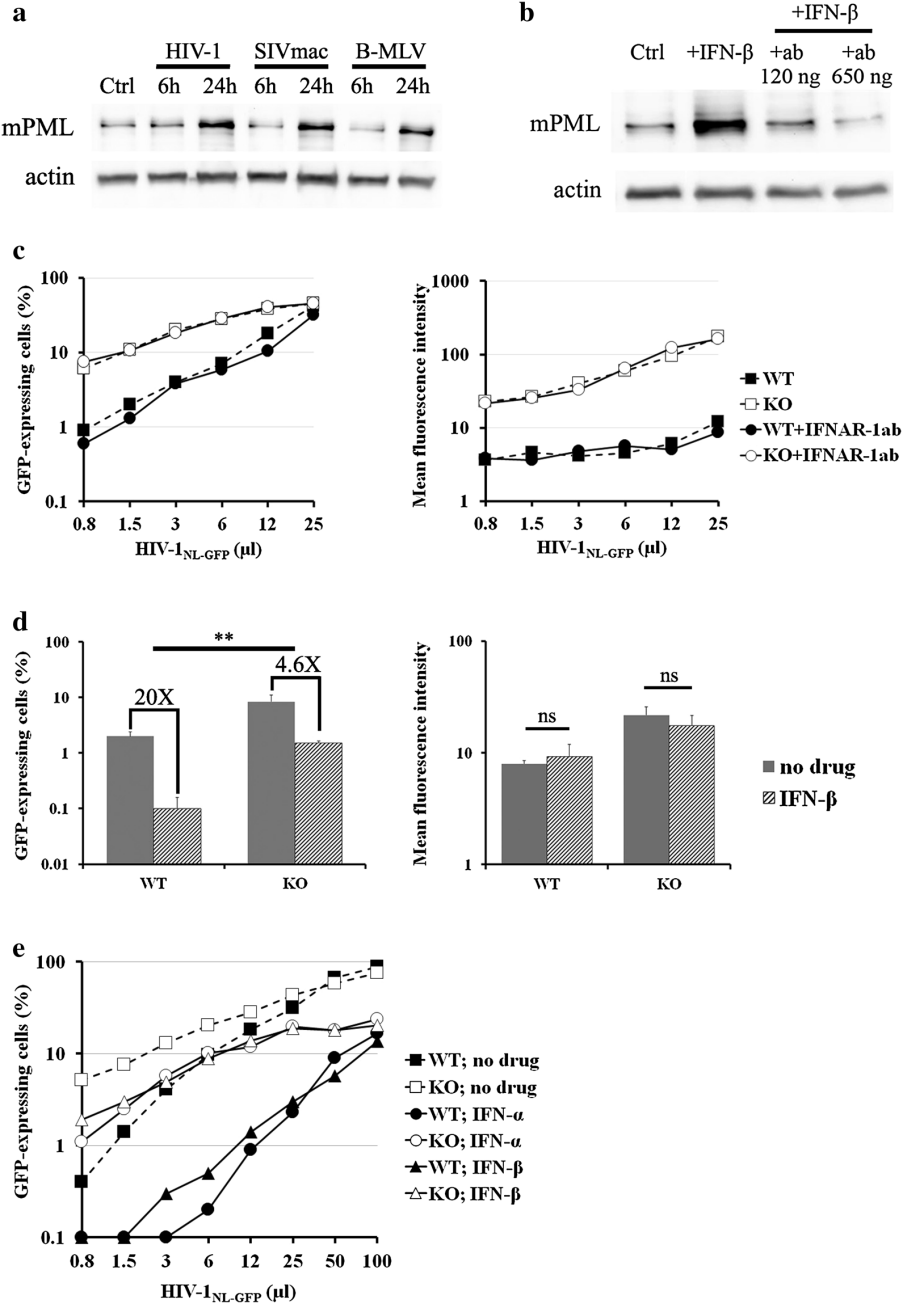
HIV-1 restriction by mPML does not require a type I IFN-induced antiviral state, but efficient IFN-induced inhibition of HIV-1 in MEFs requires PML. **a** WB analysis of infection-induced up-regulation of PML in MEFs. WT MEF cells were infected with HIV-1NL-GFP, SIV_mac-GFP_, or B-MLVGFP at an MOI of 1. Protein extracts were analyzed by WB at 6 or 24 h post infection, along with a no-infection control, using an anti-mPML monoclonal antibody (*upper panel*). Actin was analyzed as a loading control. **b** Expression of mPML was analyzed in WT MEFs *left* untreated (Ctrl), treated with IFN-β alone, or treated with a blocking antibody against IFNAR-1 at two different doses to block IFN-β-induced signal transduction prior to IFN-β treatment. mPML was detected using a monoclonal antibody (*upper panel*). Actin was analyzed as a loading control. **c** Blocking the IFN-I receptor does not alter HIV-1_NL-GFP_ restriction by PML. PML-KO and WT MEFs were treated with the anti-IFNAR-1 antibody, or with PBS as a control, and were then infected with increasing doses of HIV-1_NL-GFP_. The percentage of infected cells (*left panel*) and GFP MFI (*right panel*) were assessed 2 days later by FACS. **d** The effects of IFN-I and PML on the antiviral state. PML-KO and WT MEFs were treated with IFN-β for 16 h prior to infection with HIV-1_NL-GFP_. The percentage of infected cells (*left panel*) and GFP MFI (*right panel*) were assessed 2 days later by FACS. The values represent the means of three independent infections with standard deviations (**P < 0.01, two-tailed Student’s *t* test; *ns* non-significant). **e** Virus dose-dependent analysis of the role of PML in IFN-induced HIV-1 restriction. WT and PML-KO MEFs were treated with either IFN-α (500 U/ml) or IFN-β (100 U/ml) for 16 h, followed by infection with increasing doses of HIV-1_NL-GFP_. The percentage of infected cells was assessed 2 days later by FACS

The hPML expression levels can be altered during infection with some viruses, such as HSV-1, HCMV and Epstein–Barr virus (EBV) [47]. Interestingly, we observed a significant increase in the levels of mPML expression in response to infection of MEFs with the HIV-1_NL-GFP_, SIV_mac-GFP_, and B-MLV_GFP_ vectors (Fig. 6a), suggesting an interferon-dependent mechanism. Accordingly, the expression of PML is known to be increased in response to both type I and II IFNs [9, 48]. IFNs might be relevant to the observed HIV-1 restriction phenotype in MEFs in two ways. First, IFN treatment-mediated antiviral activities might be dependent on PML. Second, PML could indirectly interfere with HIV-1 infection and/or transcription by upregulating the production of type I IFN. To test the latter hypothesis, IFN-induced signaling in MEFs was prevented by using a blocking antibody against the mouse IFN-alpha/beta receptor subunit 1 (IFNAR-1) [49]. The efficacy of this antibody was determined by treating WT MEFs with murine IFN-β in the presence or absence of the blocking antibody and then measuring the levels of mPML expression by WB (Fig. 6b). The results show that, as expected, PML levels were greatly increased by IFN-β treatment, whereas treatment with the anti-IFNAR-1 antibody abrogated this effect in a dose-dependent fashion. Next, WT and PML-KO MEFs were treated with the blocking antibody (650 ng per 20,000 cells) prior to infection with increasing doses of HIV-1 (Fig. 6c). Compared with control untreated cells, inhibition of IFN-induced signal transduction did not modulate HIV-1 infectivity (Fig. 6c, left panel) nor HIV-1 LTR-driven GFP expression levels (Fig. 6c, right panel) in either WT or PML-KO MEFs. These results indicate that PML-mediated restriction of HIV-1 in MEFs does not require activation of IFN-mediated pathways, even though PML itself is upregulated by IFNs.

To test whether PML is important for IFN-mediated antiviral activity, we treated both WT and PML-KO MEFs with IFN-β for 16 h, then challenged them with HIV-1_NL-GFP_. As shown in Fig. 6d (left panel), treatment with IFN-β led to a 20-fold reduction in the percentage of infected cells in the presence of PML compared to only a 5-fold decrease in infectivity in PML-KO cells. However, IFN-β treatment did not modify the GFP MFI in either WT or PML-KO cells (Fig. 6d, right panel). To analyze further the importance of PML in IFN-β-mediated inhibition of HIV-1, we treated WT and PML-KO cells with murine IFN-α or IFN-β and then infected them with increasing doses of HIV-1_NL-GFP_. We found that IFN treatment reduced the infectivity of HIV-1 by up to ~100-fold at low virus doses in WT cells. However, the inhibitory effect of IFNs was significantly more modest (up to ~10-fold) in PML-KO cells (Fig. 6e). Therefore, our data support a model where type I IFNs inhibit HIV-1 through mechanisms that partially involve the PML-mediated inhibition of early replication stages but are not relevant to the inhibition of LTR-driven gene expression. Thus, PML-mediated antilentiviral functions can be both induced and constitutive.

### Human PML expression induces restriction of HIV-1 and SIV_mac_ in MEFs

**Fig. 7:**
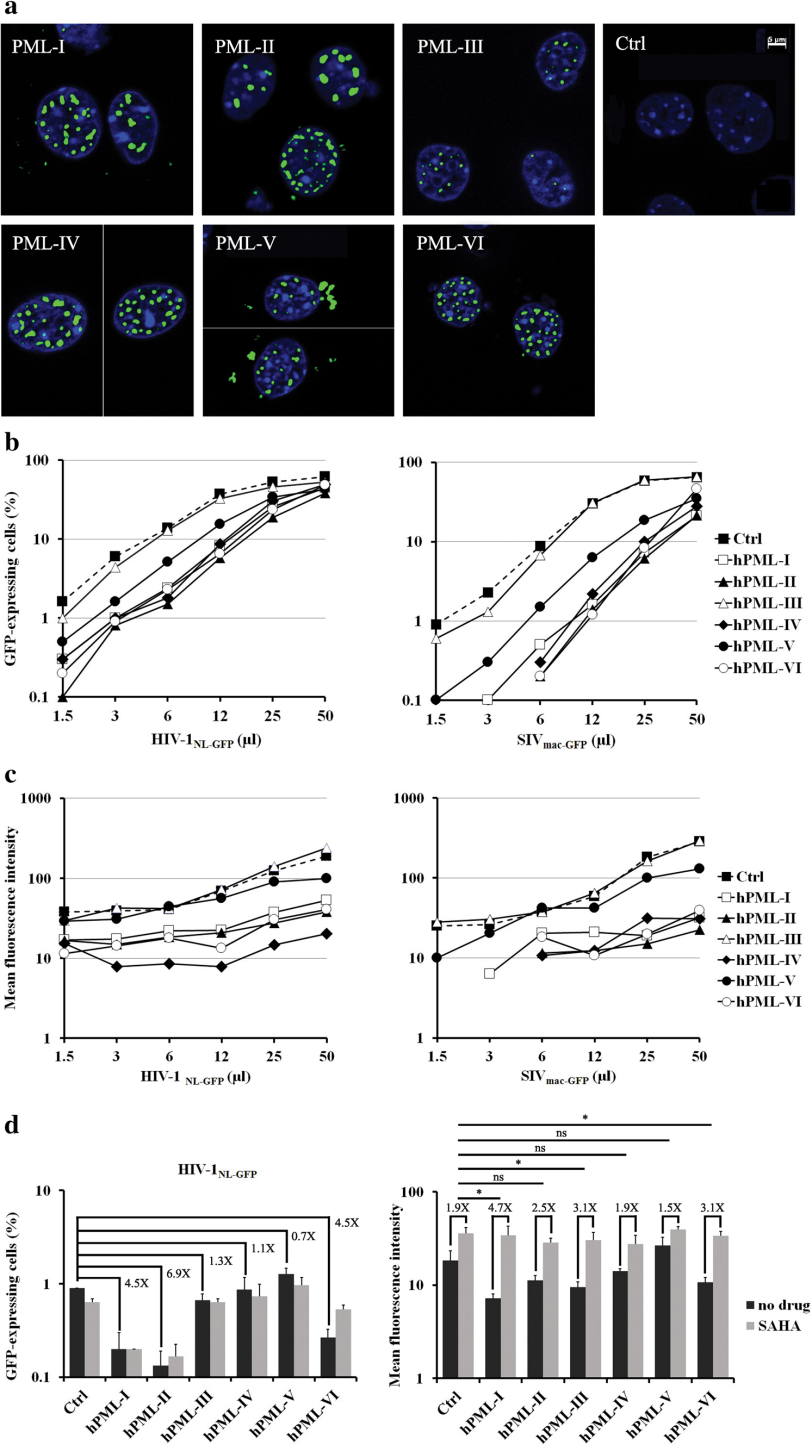
Expression of hPML isoforms in MEFs restricts HIV-1 and SIV_mac_. **a** Immunofluorescence staining of hPML in PML-KO MEFs stably transduced with FLAG-tagged hPML isoforms I to VI. Images are representative of multiple observations. hPMLs were stained with an anti-FLAG antibody (*green*) and nuclear DNA was stained using Hoechst 33342 (*blue*). *Scale bar* 5 µm. **b** Effects of hPML isoforms on HIV-1 and SIV_mac_ infectivity. PML-KO cells transduced with individual hPML isoforms were infected with increasing doses of HIV-1_NL-GFP_ (*left*) or SIV_mac-GFP_ (*right*). The percentage of infected cells was measured 2 days later by FACS. **c** Effects of hPML isoforms on HIV-1 and SIV_mac_ LTR-driven GFP expression. GFP MFI values are shown for the experiments in **b**. **d** Effects of SAHA on hPML-dependent restriction of HIV-1 infectivity and LTR-driven GFP expression. MEF cells transduced with the individual hPML isoforms were infected with HIV-1_NL-GFP_ at a CRFK MOI of 0.1. Ten days later, the cells were treated with either DMSO (no drug control) or SAHA for 48 h, followed by FACS. The percentage of infected cells (*left panel*) and GFP MFI (*right panel*) were assessed. The values represent the means of three independent experiments with standard deviations. The SAHA-dependent fold-increase in GFP MFI was compared between cells transduced with individual hPML isoforms and those transduced with the empty vector (Ctrl) using the two-tailed Student’s *t* test. The calculated P values are indicated on the *graph*. *ns* non-significant

To investigate the isoform specificity and the cellular context specificity of the restriction of HIV-1 by hPML, we expressed several hPML isoforms in PML-KO MEFs. We stably transduced FLAG-tagged versions of all six nuclear hPML isoforms (isoforms I to VI) [50] individually into these cells. The cells were selected in puromycin to eliminate untransduced cells. Immunofluorescence staining of the transduced MEF cells using an anti-FLAG antibody indicated that the different hPML isoforms were expressed in nuclei (Fig. 7a), though some cytoplasmic staining was detected for PML-V. WB analyses confirmed that all isoforms were expressed, albeit at various levels, with isoforms III, IV and V being expressed at apparently lower levels (see Additional file 2). The cells were then infected with increasing doses of HIV-1_NL-GFP_ or SIV_mac-GFP_ (Fig. 7b). HIV-1 was ~5- to 10-fold less infectious in PML-KO MEFs expressing hPML-I, II, IV and VI, compared with the empty vector-transduced control cells. PML-V had a more modest effect and PML-III did not impede HIV-1 infection (Fig. 7b, left panel). Transduction of hPML-I, II, IV, and VI in PML-KO MEFs also reduced the infectivity of SIV_mac-GFP_, by up to 44-fold (Fig. 7b, right panel). Similar to HIV-1_NL-GFP_, SIV_mac-GFP_ infectivity was modestly inhibited by hPML-V and was not affected by hPML-III. HIV-1 LTR-driven gene expression was assessed by measuring the GFP MFI in the infected cells. We observed that hPML-I, II, IV, and VI caused a reduction in GFP levels in MEFs infected with HIV-1_NL-GFP_ or SIV_mac-GFP_, while hPML-III and V had no effect (Fig. 7c). These data show that hPML can restrict HIV-1 and SIV_mac_ in MEFs but in an isoform-specific fashion. In addition, hPML-V decreased HIV-1 infectivity but had no effect on HIV-1 LTR-driven GFP expression levels, implying that these two restriction mechanisms are genetically separable.

We next tested whether SAHA treatment would specifically rescue HIV-1 LTR-driven GFP expression in MEF cells expressing human PML isoforms. PML-KO MEFs stably expressing hPML isoforms I to VI were infected with low doses of HIV-1_NL-GFP_ and kept in culture for 10 days. The cells were then treated with either 5 µM of SAHA or with DMSO as a control for 48 h, followed by FACS analysis. We observed that expression of hPML isoforms I, II and VI led to a decrease in HIV-1_NL-GFP_ infectivity, although the magnitude of this inhibition was slightly smaller than what was observed 2 days post-infection. hPML-IV and V did not significantly decrease HIV-1_NL-GFP_ infectivity, as seen at this time-point (Fig. 7d, left panel), perhaps due to the fact that these human isoforms delayed infection with HIV-1 rather than disrupting it entirely. hPML-I, II, III and VI caused a reduction in GFP MFI in these conditions (Fig. 7d, right panel), but this effect was also smaller than we had observed at 2 days post-infection (Fig. 7c). As expected, SAHA treatment had no significant effect on the infectivity of HIV-1_NL-GFP_ in PML-KO MEFs expressing the various hPML isoforms (Fig. 7d, left panel). SAHA slightly increased (1.9-fold) the GFP MFI in the control cells (Fig. 7d, right panel), a result similar to what we had observed before (Fig. 5). The effect of SAHA was significantly greater in cells stably expressing hPML-I, III and VI (Fig. 7d, right panel), suggesting that the mechanism of inhibition of GFP expression by these isoforms was transcriptional silencing, similar to what we had demonstrated with mPML. Taken together, these data suggest that hPML can mediate the two inhibitory phenotypes also observed with mPML, although in an isoform-specific, cellular context-specific manner.

## Discussion

The results from this study show that PML can interfere with at least two distinct steps in the replication of HIV-1 and other lentiviruses. The first block to replication occurs at early post-entry stages and was seen in both MEF and SupT1 cells, although the magnitude of the restriction was significantly higher in the murine cells. The existence of an early post-entry block to HIV-1 replication in murine cells has long been known [51, 52]. This restriction of HIV-1 infection was seen in all murine cell types analyzed by these investigators, although it was stronger in lymphocytes compared to fibroblasts [53]. On the basis of viral DNA analyses, the block was found to occur prior to integration [53–55], consistent with recent results from other groups [22, 23]. Here we show that in addition to HIV-1, the early post-entry replication of two other lentiviruses, SIV_mac_ and EIAV, is restricted in murine MEFs. Our results indicate that PML is required for this early phase of restriction to occur. Another team recently reported that the PML body component Daxx was involved in the PML-mediated inhibition of HIV-1 [23], though this finding was contradicted in a report from another group [22]. We find that the PML-dependent restriction of early-stage HIV-1 infection was increased by treatment with IFN-α or IFN-β, which suggests that PML is relevant to the intrinsic cellular defenses against retroviral infections. Type I IFN treatment increased expression of mPML itself (Fig. 6), yet mPML overexpression was not sufficient to increase the restriction of incoming HIV-1 or SIV_mac_ in MEFs (Fig. 4). These various observations are consistent with a model thereby IFN-I inhibits HIV-1 in mouse cells by increasing the expression of a restriction factor that acts downstream of PML and directly targets incoming HIV-1. Along these lines, restriction of HIV-1 appeared to be saturable in several of our experiments, supporting a model where an antiviral effector is present in limiting concentrations, which is not consistent with PML being this effector. Also in support of an indirect effect of PML is the fact that HIV-1 infection of MEFs was inhibited at reverse transcription, a step that takes place in the cytoplasm while PML is predominantly nuclear. Taken together, these observations suggest that PML promotes the restriction of multiple lentiviruses by activating a downstream effector whose identity and viral target(s) remain to be determined. Interestingly, PML was recently found to be involved in the transcriptional activation of interferon-stimulated genes following treatment with IFN-I [56], supporting the idea that PML plays an activating role upstream of innate immune effectors.

In addition to its effects on early stage viral replication, PML also caused transcriptional silencing of HIV-1 in MEFs, a result consistent with previous observations that HIV-1 transcription was low in murine cells, even in the presence of human cyclin T1 (hCycT1) [57]. Unlike the restriction of early stages of replication, the repression of HIV-1 gene expression was not enhanced by IFN-I treatment. Therefore, although both inhibitory mechanisms are dependent upon the presence of PML, they are differentially regulated. We found no evidence that hPML repressed HIV-1 LTR-driven gene expression in SupT1 cells. However, transfer of some hPML isoforms (hPML-I, II, IV and VI and to a lesser extent hPML-V) in PML-KO MEFs fully reconstituted the restriction activities, supporting a conserved role for mPML and hPML. This discrepancy may result from the fact that the establishment of latency in human cells may be rare and may occur only in specific conditions, whereas the HIV-1 promoter is constitutively repressed in murine cells. Recently, two different teams used cell lines belonging to the “J-Lat” series, which are human T cell lines containing integrated but transcriptionally silent copies of an HIV-1-derived vector, to investigate whether PML and PML bodies have a role in latency. Both teams found that latent HIV-1 could be reactivated by treatment with the PML inhibitor arsenic trioxide (As_2_O_3_) [32, 58]. Lusic and collaborators also observed that PML depletion similarly reactivated HIV-1 in the J-Lat 9.2 clone [32]. These results show that PML is required for the maintenance of transcriptional latency in these models, but they do not address the question of whether it is involved in the establishment of latency. MEFs and possibly other murine cell types in which HIV-1 transcription is constitutively repressed may provide valuable investigatory tools to identify the factors controlling the establishment and maintenance of viral latency and persistence.

As_2_O_3_ has been shown to interfere with several retroviral restriction pathways over recent years, including TRIM5α [20, 59], TRIMCyp [60], APOBEC3G [61], Lv4 [62] and possibly SAMHD1 [63]. As_2_O_3_ is unlikely to directly inhibit those various restriction effectors. Therefore, the most straightforward explanation is that it acts upstream, by interacting with a factor that controls the global antiviral state of the cell. Clues that PML might be this factor come from imaging and biochemical studies that used fluorescent and biotin-labeled analogs of As_2_O_3_. These studies strongly suggested that PML was the major and perhaps the sole cellular target for this drug [64, 65]. As_2_O_3_ promotes PML oligomerization, resulting in increased SUMOylation and ubiquitination, followed by proteasome-dependent degradation [64]. The picture emerging from these and other studies [56] is that PML upregulates antiretroviral effectors that target viral replication at several steps. Changes in the expression patterns of these downstream effectors might explain the cellular context specificity observed for the effects of PML expression on HIV-1.

## Conclusions

Taken together, our observations suggest that PML broadly upregulates the activity of innate antiviral effectors, through mechanisms that are yet to be dissected. It has been suggested that the PML inhibitor As_2_O_3_ could be tested as a pharmacological agent to counter HIV-1 latency in humans [32, 58]. However, this study and previous ones [20, 23] show that targeting PML might enhance the early stages of HIV-1 replication by removing PML-controlled antiviral activities. Thus, As_2_O_3_ and other compounds targeting PML likely involve a tradeoff between inhibition of latency and inhibition of innate immune mechanisms. Our results are also relevant to the development of murine models for HIV-1. Despite multiple attempts at introducing key human positive factors in murine cells, such as hCD4, hCCR5, hCycT1 or hCRM1 [64, 66, 67], murine cells remain non-permissive to HIV-1. Removing the endogenous mPML in the context of murine cells expressing key human factors might support HIV-1 propagation. The availability of PML knockout mice for crossing experiments [40] might finally open the door to the long sought-after human tissue-free murine model for AIDS.

## Methods

### Cell culture

Immortalized PML-KO and WT MEFs were a generous gift from Pier P. Pandolfi [40]. Crandell-Rees feline kidney (CRFK), human embryonic kidney (HEK) 293T and MEF cells were maintained in Dulbecco’s modified Eagle’s medium (DMEM; HyClone, Thermo Scientific, USA). SupT1 cells were maintained in RPMI 1640 (HyClone). All culture media were supplemented with 10 % fetal bovine serum (FBS) and penicillin/streptomycin (HyClone).

### Plasmids, transfections and transductions

To transduce mPML using a retroviral vector, total RNA was extracted from WT MEF cells using Trizol (Invitrogen, Carlsbad, CA) according to the manufacturer’s instructions. First-strand cDNA synthesis was conducted using 2 µg of total RNA, random hexamers and the SuperScript III first-strand synthesis kit (Invitrogen) following treatment with DNase I (NEB), as described in the manufacturer’s protocol. Mouse PML (mPML) cDNA was then amplified by PCR using the oligodeoxynucleotide (ODN) primers whose sequences are provided in the Additional file 3. The resulting 2.65-kb cDNA fragment was cut with BamHI-MfeI and then inserted into the MLV-based retroviral vector pMIP [43], and cut with BglII and EcoRI, yielding pMIP-mPML. The cloned PML cDNA was sequenced and determined to be a variant of isoform 2 (GenBank accession No. KJ650238). To transduce N-terminally FLAG-tagged versions of hPML isoforms I to VI using a retroviral vector, individual isoforms were PCR amplified from the corresponding pLNGY-hPML constructs generously provided by R. D. Everett [50], using the ODNs shown in Table A1, and cloned into pMIP, which had been cut with BglII-EcoRI, yielding pMIP-hPML-I to -VI.

Retroviral vectors expressing mPML or hPML were prepared by cotransfection of 293T cells plated at 70 % confluency in 10 cm dishes with 10 µg of pMIP-m(h)PMLs together with 5 µg of pMD-G [68] and 10 µg of pCl-Eco [69] using polyethylenimine (PEI; Polyscience, Niles, IL). Virus-containing supernatants were collected 2 days later and clarified by low-speed centrifugation, as described previously [20, 70]. Stable mouse or human PML-expressing MEFs were obtained by spinfection of 2 × 10^5^ cells with 2 ml of retroviral vector-containing supernatants for 50 min at 400×*g* in the presence of 8 µg/ml polybrene (Sigma-Aldrich, MO, USA) [71] and followed by a 24 h incubation at 37 °C. In order to eliminate the non-transduced cells, puromycin (Calbiochem, CA, USA) was then added to the cultures at a final concentration of 2 µg/ml for 5 days. The relevant “empty” (non-PML-expressing) vector was transduced as a control in all experiments.

To produce GFP-expressing retroviral vectors, 293T cells were seeded in 10 cm culture dishes and transiently cotransfected as described above. The plasmids used were as follows: pMD-G, pCNCG and pCIG3-B to produce B-MLV_GFP_ [72, 73]; pMD-G and pNL-GFP to produce HIV-1_NL-GFP_ [20, 33]; pMD-G and pSIV_mac_239_GFP_ to produce SIV_mac-GFP_ [34]; or pONY3.1, pONY8.0 and pMD-G to produce EIAV_GFP_ [74]. The supernatants were replaced with fresh medium after 6 h and the retroviruses were harvested 24 h later. The retroviruses were clarified by centrifugation at 3000 rpm and stored in aliquots at −80 °C. The viral stocks were titered by serial dilution on CRFK cells.

### RNA interference

ODNs were designed to create pAPM-based, shRNA-expressing constructs targeting hPML, as described previously [75, 76]. The shRNAs expressed targeted the following sequences, present in all hPML isoforms: shPML1, AAGATGCAGCTGTATCCAAGA; shPML2, GCAAGACCAACAACATCTTCT; shPML3, GCACACGCTGTGCTCAGGATG. The full sequences of the ODNs used to generate these constructs are provided in Additional file 3. SupT1 cells were stably transduced with shRNAs targeting hPML or Luciferase as a control via lentiviral gene transfer. Briefly, lentiviral vectors were prepared by cotransfection of HEK293T cells with 10 µg of either pAPM-shLuc [76] or pAPM-shPML1-3, together with 5 µg of pMDG and 10 µg of pΔR8.9 [68], as described above. The viral supernatants were used for transduction of shPMLs into SupT1 cells, as detailed above. Stably transduced cells were selected by addition of 5 µg/ml puromycin to the medium at 2 dpi and for 5 days.

### Antibodies and WB analyses

The cells were lysed at 4 °C in RIPA lysis buffer (1 % NP40, 0.5 % deoxycholate, 0.1 % SDS, 150 mM NaCl, 50 mM Tris–HCl pH 8.0). The lysates were subjected to SDS–polyacrylamide gel electrophoresis, followed by WB analysis using mouse anti-mPML mAb (36-1-104, Enzo life sciences, NY, USA), rabbit polyclonal anti-hPML (H-238, Santa Cruz, TX, USA), anti-FLAG (Cell Signaling, MA, USA), or anti-β-actin antibody (Sigma, MI, USA). The p24 capsid protein of HIV-1 (CA, p24) was detected using a mouse monoclonal antibody (clone 183, AIDS Research and Reference Reagent Program Cat. No. 3537).

### Immunofluorescence microscopy

PML-KO cells stably transduced with FLAG-tagged hPML-I to VI isoforms or WT MEFs were seeded on glass coverslips placed in 3.5-cm wells. After 24 h, the cells were permeabilized and fixed for 10 min in Triton X-100/4 % formaldehyde at room temperature (RT), followed by four washes with PBS. The cells were then treated with 10 % goat serum (Sigma) for 30 min at RT followed by 4 h of incubation with antibodies against FLAG (Sigma, 1:150) or hPML (Santa Cruz, 1:150) or mPML (Enzo Life Sciences, 1:150) in 10 % goat serum at RT. They were then washed four times with PBS and fluorescently stained with Alexa Fluor 488-conjugated goat anti-mouse (Molecular Probes, Eugene, OR) diluted 1:100 in 10 % goat serum for 1 h at RT. The cells were then washed 4 times with PBS before mounting in Vectashield (Vector Laboratories, Peterborough, UK). Hoechst 33342 (0.8 μg/ml; Molecular Probes) was added along with the penultimate PBS wash to reveal DNA. Z-stacks were acquired on an AxioObserver Microscope (Carl Zeiss Canada, Toronto, ON) equipped with the Apotome module, and the median optical slice of each Z-stack was analyzed.

### Pharmacological treatments

SAHA (Sigma-Aldrich) was resuspended in DMSO and used at a final concentration of 5 µM for 48 h prior to flow cytometric analysis. Recombinant murine IFN-α (11200-2) and IFN-β (12405-1) were obtained from PBL Interferon Source (NJ, USA) and added to the cells 16 h prior to infection with retroviruses. 24 h after infection, the supernatants were replaced with fresh IFN-containing medium. To block the extracellular domain of the IFN-I receptor in MEFs, the cells were treated with purified anti-mouse IFNAR-1 antibody (MAR1-5A3, BioLegend, UK), at a concentration of 650 ng per 20,000 cells, 1 h prior to infection with HIV-1_NL-GFP_. Where applicable, the supernatants were replaced with fresh drug-containing medium 24 h after infection.

### Viral challenges and flow cytometric analysis

The cells were seeded into 24-well plates at 2 × 10^4^ cells/well (MEF) or 1 × 10^5^ cells/well (SupT1) and infected the following day with GFP-expressing retroviral vectors. MEF cells were trypsinized at 2 dpi and fixed in 3 % formaldehyde (Fisher Scientific, MA, USA). The percentage of GFP-positive cells and MFI were then determined by analyzing 10^4^ cells on a FC500 MPL cytometer (Beckman Coulter, CA, USA) using the CXP Software (Beckman Coulter). MFI analysis was restricted to the GFP-positive cells.

### Quantitative real-time PCR

The late RT products, 2-LTR circles, and HIV-1_NL-GFP_ mRNA expression levels in infected cells were measured by either qPCR or qRT-PCR using the Stratagene Mx3000P system (Agilent, CA, USA). The cells were plated in 12-well plates at 3 × 10^5^ cells/well and infected with HIV-1_NL-GFP_. The retrovirus was pretreated with 20 U/ml DNase I (NEB) for 1 h at 37 °C and control infections were performed in the presence of 80 µM nevirapine (Sigma), as described previously, to demonstrate the absence of carry-over contaminating plasmid DNA [77]. Total cellular DNA was collected after 6 h of infection (late RT products) or 6 h of infection followed by 18 h incubation in virus-free medium (2-LTR-circles) using the QIAamp DNA mini kit (Qiagen, CA, USA). Sequence data for the ODNs used in the PCR reactions (GFP forward and reverse, 2-LTR circles forward and reverse, actin forward and reverse) is provided in Additional file 3. The reactions contained 1x SensiFast SYBR Lo-ROX mix (Bioline, UK), 400 nM forward and reverse primers, and 5 µl template (150–400 ng) in 20 µl final volume. After 3 min incubation at 95 °C, 40 cycles of amplification were performed as follows: 5 s at 95 °C, 10 s at 62 °C (GFP) or 65 °C (2-LTR), 15 s at 72 °C.

For qRT-PCR, total RNAs were purified from infected or uninfected MEFs using the AllPrep RNA/Protein kit (Qiagen). Reverse transcription of 200 ng of each RNA sample followed by real time PCR were performed in a final volume of 20 µl using the SensiFAST SYBR Lo-ROX One-Step kit (Bioline) according to the manufacturer’s instructions. The primer sets to detect GFP and actin in the PCR reactions were as mentioned above. The reaction conditions were: 48 °C for 30 min, 95 °C for 10 min, 40 cycles of amplification: 95 °C for 15 s, 60 °C for 30 s. Primers were validated by performing a standard curve and through dissociation curves analysis. Plasmid copy numbers dilutions ranging from 5.5 × 10^5^ down to 14 were used for the GFP standard curve. Results were then analyzed with the MxPro software (Agilent). Absolute counts were determined using the equation of the standard curve log(y) = ax + b where copy number was 10^((Ctsample−b)/−a)^.

Each PCR was performed in duplicate and the threshold cycle (C_t_) was determined using the MxPro software (Agilent). In each experiment, a standard curve was run in duplicate, ranging from 300 to 3 × 10^5^ copies plus a no-template control. The levels of HIV-1 transcript were normalized to those of GAPDH, which was quantified in parallel as an endogenous control.

## Additional files

10.1186/s12977-016-0253-1 Relative quantification of HIV-1 transcription.
10.1186/s12977-016-0253-1 Western blot analysis of hPML isoforms overexpressed in PML-KO MEFs.
10.1186/s12977-016-0253-1 Sequences of primer ODNs used in this study.
