# Supplementary material for: The interferon-induced antiviral protein PML (TRIM19) promotes the restriction and transcriptional silencing of lentiviruses in a context-specific, isoform-specific fashion
Source: Retrovirology. 2016 Mar 22;13:19. doi: 10.1186/s12977-016-0253-1 (PMC4802722; doi:10.1186/s12977-016-0253-1)
Supplement: Supplementary file 1 — 10.1186/s12977-016-0253-1 Relative quantification of HIV-1 transcription. [file 12977_2016_253_MOESM1_ESM.pdf]

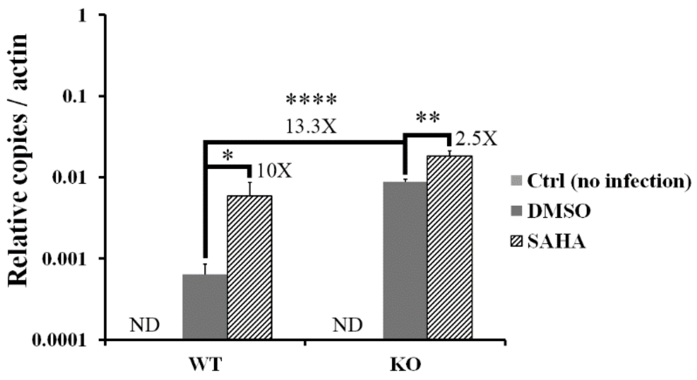

Additional file 1. Relative quantification of HIV-1 transcription.

WT or PML-KO MEFs were challenged with HIV-1_{NL-GFP} in triplicate (CRFK MOI = 0.01). Ten days later, the cells were treated with either DMSO or SAHA for 48 h. Total RNAs were then purified from the cells and the levels of GFP and actin transcripts were determined by qRT-PCR. Data are presented as the ratios of GFP compared to actin mRNAs. Total RNAs from uninfected cells were used as negative control. The values represent the means of three independent experiments with standard deviations (*P< 0.05, **P< 0.01, ****P< 0.0001, two-tailed Student's *t*-test). ND, not detected.
