# Supplementary material for: The interferon-induced antiviral protein PML (TRIM19) promotes the restriction and transcriptional silencing of lentiviruses in a context-specific, isoform-specific fashion
Source: Retrovirology. 2016 Mar 22;13:19. doi: 10.1186/s12977-016-0253-1 (PMC4802722; doi:10.1186/s12977-016-0253-1)
Supplement: Supplementary file 2 — 10.1186/s12977-016-0253-1 Western blot analysis of hPML isoforms overexpressed in PML-KO MEFs. [file 12977_2016_253_MOESM2_ESM.pdf]

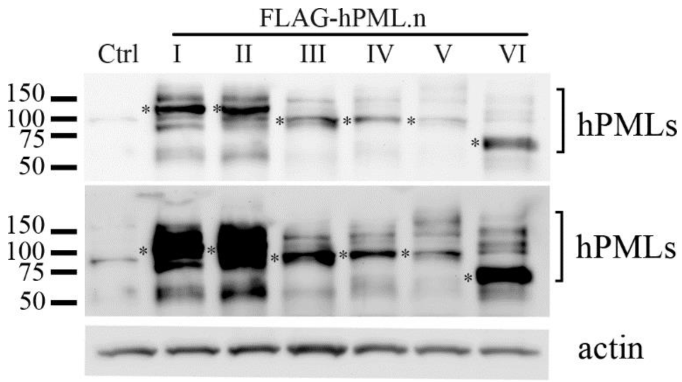

Additional file 2. Western blot analysis of hPML isoforms overexpressed in PML-KO MEFs.

Cells were stably transduced with hPML isoforms I to VI. The upper panel shows a WB analysis of hPML expression using a monoclonal antibody (H-238). The bands labeled with asterisks correspond to the expected isoforms according to their sizes. The heavier bands are likely to be SUMO-modified PML. The lower panel shows the same blot reprobed using an anti-FLAG antibody. Actin was used as a loading control.
