# Supplementary material for: The interferon-induced antiviral protein PML (TRIM19) promotes the restriction and transcriptional silencing of lentiviruses in a context-specific, isoform-specific fashion
Source: Retrovirology. 2016 Mar 22;13:19. doi: 10.1186/s12977-016-0253-1 (PMC4802722; doi:10.1186/s12977-016-0253-1)
Supplement: Supplementary file 3 — 10.1186/s12977-016-0253-1 Sequences of primer ODNs used in this study. [file 12977_2016_253_MOESM3_ESM.pdf]

Mouse PML forward	5’CACGGATCCACCATGGAAACTGAACCAGTTTCC 3’
Mouse PML reverse	5’ATCCAATTGCTAGGCCAGGCATCCCTTACTTTCAGC 3’
Human PML forward	5’AATTAGTCGACGGATCCACCATGGACTACAAAGACGATG ACGACAAGGAGCCTGCACCCGCCCGATC 3’
Human PML-I reverse	5’ATCCAATTGTCAGCTCTGCTGGGAGGCCCTCTCTG 3’
Human PML-II reverse	5’ATCGAATTCTCAGAGGCCTGCTTGACGGGCGCCTG 3’
Human PML-III reverse	5’ATCCAATTGTCAGCGGGCTGGTGGGGAGGCCAAG 3’
Human PML-IV reverse	5’AGCCAATTGCTAAATTAGAAAGGGGTGGGGGTAGC 3’
Human PML-V reverse	5’AGCCAATTGTCAATGCCTCACTGGAAAATTCCCCAG 3’
Human PML-VI reverse	5’ATCCAATTGTCACCACAACGCGTTCCTCTCCCTACC 3’
shPML1	5’TGCTGTTGACAGTGAGCGCAAGATGCAGCTGTATCCAAG ATAGTGAAGCCACAGATGTATCTTGGATACAGCTGCATCTT TTGCCTACTGCCTCGGA3’
shPML2	5’TGCTGTTGACAGTGAGCGAGCAAGACCAACAACATCTTC TTAGTGAAGCCACAGATGTAAGAAGATGTTGTTGGTCTTGC GTGCCTACTGCCTCGGA3’
shPML3	5’TGCTGTTGACAGTGAGCGCGCACACGCTGTGCTCAGGAT GTAGTGAAGCCACAGATGTACATCCTGAGCACAGCGTGTG CATGCCTACTGCCTCGGA3’
GFP forward	5’GACGACGGCAACTACAAGAC3’
GFP reverse	5’CGGATCTTGAAGTTCACCTTG3’
2-LTR circles forward	5’AACTAGGGAACCCACTGCTTAAG3’
2-LTR circles reverse	5’TCCACAGATCAAGGATATCTTGTC3’
Actin forward	5’CCTCCCTGGAGAAGAGCTA3’
Actin reverse	5’ACGTCACACTTCATGGA3’

Additional file 3. Sequences of primer ODNs used in this study.
